# Potentiel zoonotique de la brucellose des mammifères marins

**DOI:** 10.48327/mtsi.v4i1.2024.489

**Published:** 2024-02-19

**Authors:** Hédia ATTIA EL HILI, Kaouthar MAATOUK

**Affiliations:** 1 Centre national de veille zoosanitaire, Tunis, Tunisie; 2 Institut national des sciences et technologies de la mer, Monastir, Tunisie

**Keywords:** Brucellose, Zoonose, Mammifères marins, Sources, Transmission, Exposition, Brucellosis, Zoonosis, Marine mammals, Sources, Transmission, Exposure

## Abstract

**Introduction:**

Durant les deux dernières décennies, la brucellose chez les mammifères marins (cétacés et pinnipèdes) a émergé d'une manière très significative. À l'heure actuelle *Brucella ceti* et *Brucella pinnipedialis* sont les deux espèces reconnues chez ces animaux, mais les données disponibles à leur sujet sont encore limitées. Plusieurs génotypes ont été identifiés. Des études ont mis en évidence des différences de pathogénicité ou de tropisme d'organe selon le type de séquence (ST) de *B. ceti* chez les cétacés. L'appréciation du potentiel zoonotique de ces bactéries repose sur l'identification des sources essentielles d'introduction et de propagation des brucelles marines ainsi que des facteurs d'exposition des mammifères marins et des humains.

**Revue bibliographique:**

Cet article représente une synthèse bibliographique des références publiées sur la brucellose chez les mammifères marins, les caractéristiques des espèces de *Brucella*, leurs sources, leurs modes de transmission, ainsi que les risques de contamination des animaux et des humains.

**Conclusion:**

La gravité de cette maladie demeure inconnue en raison du manque d'estimation de sa prévalence chez les mammifères marins. Le nombre de cas humains suspectés est encore très limité. Mais, par analogie avec d'autres germes du genre *Brucella* responsables d'avortement chez les ruminants et d'un état fébrile et algique dans l'espèce humaine, des mesures de prévention s'imposent en pensant aux activités ou aux métiers exposés. L'augmentation significative du nombre d’échouages couplée à une haute séroprévalence chez certaines espèces de mammifères marins doivent être prises en compte pour les personnes en contact direct ou indirect avec des mammifères marins. Une épidémio-surveillance continue associée à des examens post-mortem poussés (autopsie, bactériologie, séquençage…) de toutes les espèces de mammifères marins échoués permettrait d'approfondir les connaissances sur le portage de brucelles par ces animaux et sur le potentiel zoonotique des espèces marines de *Brucella*.

## Abstract (en)

**Introduction:**

Brucellosis in marine mammals (cetacean and pinnipeds) has emerged in a very significant way during the last two decades. Currently *Brucella ceti* and *Brucella pinnipedialis* are the two recognized species in marine mammals, but available information is still limited. Several genotypes have been identified, and studies on the relationship between sequence type (ST) and organ pathogenicity or tropism have indicated differences in pathogenesis between *B. ceti* sequences in cetaceans. The zoonotic potential of this disease is based on the identification of the main sources of introduction and spread of *Brucella* spp. in the marine environment as well as on the factors of exposure of marine mammals and humans to the bacteria.

**Bibliographic review:**

This article is a bibliographical review on marine mammal brucellosis, including the features, sources and transmission modes of each *Brucella* species, as well as their potential pathogenicity in animals and humans.

**Conclusion:**

Different genotypes of marine *Brucella* spp have been isolated from marine mammal species but without any evidence of pathology induced by these bacteria. Associated lesions are variable and include subcutaneous abscesses, meningo-encephalomyelitis, pneumonia, myocarditis, osteoarthritis, orchitis, endometritis, placentitis and abortion. The isolation of marine *B.* spp from marine mammal respiratory parasites associated to lung injury has raised the intriguing possibility that they may serve as a vector for the transmission of this bacterium.

The severity of marine *B.* spp remains unknown due to the lack of an estimate of the prevalence of this disease in marine mammals. The number of suspected human cases is still very limited. However, by analogy with other germs of the genus *Brucella* responsible for abortion in ruminants and for a febrile and painful state in human beings, prevention measures are essential. The significant increase in the number of strandings coupled with a high seroprevalence in certain species of marine mammals must be considered for people in direct or indirect contact with these animals. Ongoing epidemiological monitoring combined with extensive post-mortem examinations (necropsy, bacteriology and sequencing) of all species of stranded marine mammals would deepen knowledge on the zoonotic potential of marine *Brucella* species.

## Introduction

À l'instar de nombreux animaux terrestres, les mammifères marins peuvent être infectés par des agents pathogènes potentiellement zoonotiques [1, 27, 68, 69, 76] et présenter des signes cliniques de maladie, ou être porteurs asymptomatiques. Bien que les cas recensés de transmission de zoonoses soient rares, l'augmentation des contacts entre les humains et les mammifères marins (rencontres avec des dauphins « ambassadeurs », programmes de nage avec des dauphins captifs, études, programmes de recherches…) accroît le nombre potentiel de personnes exposées à ces animaux et à leurs germes. Le risque que ces agents pathogènes soient transmis à des humains et deviennent responsables de zoonoses est surtout lié au type de contact et à la durée d'exposition à ces animaux. Ainsi, les personnes chargées de l'entretien et de la gestion au quotidien de mammifères marins en captivité, comme les chercheurs et les vétérinaires qui manipulent ces animaux, courent un risque non négligeable de contracter une zoonose [44]. Les agents pathogènes zoonotiques trouvés chez les mammifères marins pourraient avoir été acquis via la contamination des eaux côtières par les eaux usées, le ruissellement et les déchets agricoles et médicaux [6]. Comme les mammifères marins sont au sommet de la chaîne alimentaire, ils peuvent ainsi servir d'indicateurs de la qualité de l'environnement. Les principales maladies des mammifères marins pouvant compromettre la santé publique sont surtout de nature bactérienne, virale, parasitaire et fongique [65, 67, 81]. Une meilleure compréhension de l’écologie de ces maladies devrait permettre d’évaluer les risques pour les personnes exposées, y compris les consommateurs se nourrissant principalement de ces animaux [6], et ainsi prévenir les risques d'une contamination humaine.

Parmi les zoonoses, la brucellose fait l'objet du présent travail [67, 68]. L'infection par *Brucella* spp., reconnue depuis longtemps comme cause d'avortement, de stérilité et de problèmes de reproduction chez les animaux domestiques, a émergé chez les cétacés et chez les pinnipèdes d'une manière très significative ces deux dernières décennies et elle est de plus en plus documentée. La vaste distribution géographique de cette zoonose au sein des mammifères marins suggère qu'elle est présente depuis longtemps, mais n'a été identifiée qu’à partir de 1994 [73]. La brucellose, chez les animaux domestiques (bovins, ovins, caprins et porcins) et chez les humains, est considérée parmi les maladies à déclaration obligatoire. Jusqu’à ce jour, le statut *Brucella* d'un pays est basé sur la situation épidémiologique chez les animaux domestiques et les espèces de brucelles marines ne sont pas prises en compte. Les pays reconnus comme indemnes de la maladie peuvent avoir identifié des *Brucella* spp. dans leurs populations de mammifères marins.

Cette étude est une revue de littérature qui aborde en particulier la présentation des *Brucella* des mammifères marins (cétacés et pinnipèdes) ainsi que les aspects épidémiologiques de l'infection associée. La consultation des bases de données PubMed, Scopus et Science Direct nous ont permis d'identifier des publications pertinentes traitant la brucellose chez ces animaux. La mise en évidence des sources essentielles d'introduction et de propagation de cette bactérie dans le milieu marin, ainsi que des facteurs d'exposition des mammifères marins et de l'humain à cette affection, permettrait d'apprécier le potentiel zoonotique de cette maladie.

## Les souches de *Brucella* marines

### Généralités

Des bactéries du genre *Brucella,* coccobacilles Gram négatif, du groupe a2 des Proteobacteria, de l'ordre des Rhizobiales et de la famille des *Brucellaceae,* sont responsables de l'infection zoonotique appelée brucellose [62]. Chez les humains elle est aussi connue sous le nom de fièvre ondulante, fièvre de Malte, de Gibraltar ou encore fièvre méditerranéenne. Des études fondées sur l'hybridation ADN/ADN ont montré que le genre *Brucella* était un genre très homogène (homogénéité supérieure à 90 % pour les différentes espèces) y compris les souches isolées des mammifères marins [80].

Ce genre rassemble une douzaine d'espèces différentes selon leurs différences de pathogénicité et leurs hôtes préférentiels [68]. Parmi ces espèces, *B. abortus, B. canis, B. melitensis, B. microti, B. neotomae, B. ovis, B. suis* sont déjà bien connues. Depuis 1994 *B. ceti et B. pinnipedialis* ont été identifiées chez des mammifères marins, respectivement à partir d'isolats de cétacés et de phoques [32, 33, 37, 73]. Les isolats de cétacés ont d'abord été désignés par *Brucella maris* [45], puis *Brucella cetacea* [11] et enfin corrigés en *Brucella ceti* [33]. Comme les isolats de pinnipèdes se sont avérés distincts de *B. ceti,* ils ont été alors nommés *Brucellapinnipedialis* [33]. Bien que les souches de *Brucella* d'animaux terrestres n'aient pas été identifiées chez les cétacés, l'inverse n'est pas vrai, et des souches de *B. ceti* ont été isolées de cas humains [55, 83].

Les bactéries *B. ceti* et *B. pinnipedialis* sont de petite taille, intracellulaires facultatives et mesurent 600 à 1500 nm de long et environ 500 à 700 nm de diamètre. Elles ne possèdent ni capsule, ni flagelle et sont non sporulées [52]. Les *Brucella* des mammifères marins se distinguent des formes terrestres notamment par leurs besoins en CO_2_, l'activité de l'uréase ainsi que la production d'H_2_S. Les deux espèces *B. ceti et B. pinnipedialis* se distinguent aussi phénotypiquement par leurs besoins respectifs en CO_2_ pour leur croissance primaire. En effet, la plupart des souches de *B. ceti* se cultivent en l'absence de CO_2_ alors que ce dernier est nécessaire à la majorité des souches de *B. pinnipedialis* [26].

Il est connu qu'en dehors de leur hôte, les *Brucella* terrestres sont très résistantes mais ne se multiplient pas. Elles peuvent ainsi persister plusieurs années dans des placentas ou avortons congelés, pendant des mois en condition humide à 10-15 °C et des heures à des températures de 45-50 °C [12]. Mais concernant les *Brucella* marines, leur résistance dans le milieu océanique reste à établir [41].

### Caractéristiques génétiques

Des études ayant utilisé des empreintes de l’élément génétique mobile IS 711 du gène *bp26* ont montré une différence entre le nombre et la distribution des copies de cet élément entre le génome des isolats marins et terrestres. En effet, les isolats provenant des mammifères marins contiennent davantage de copies [69]. La structure génétique globale de *B. ceti,* telle que la présence de deux chromosomes circulaires et l'absence de plasmides, ainsi que les caractéristiques bactériologiques générales sont en phase avec les *Brucella* pathogènes classiques des mammifères terrestres [72]. Comme les autres espèces de *Brucella, B. ceti* semble se répliquer à l'intérieur des macrophages et des trophoblastes de l'hôte et provoquer des maladies chroniques chez les cétacés [39, 40, 42]. Cependant, les mécanismes de la pathogenèse, de la virulence et de l'affinité avec l'hôte n'ont pas été étudiés. Le lipopolysaccharide lisse (LPS) a été identifié parmi les facteurs virulents potentiels de *B. ceti.* Il n'y a pas de différences significatives dans les gènes putatifs de la N-formylpérosamyle transférase entre les souches lisses isolées de mammifères marins et terrestres [5, 86].

**Figure 1:**
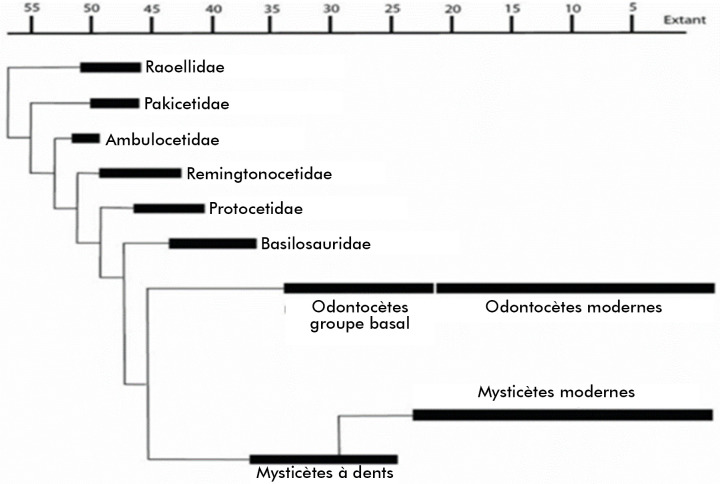
Durées de présence (en millions d'années) et phylogénie des cétacés et des artiodactyles raoellidés de l’Éocène et modernes [3] Presence duration (in millions of years) and phylogeny of Eocene and modern cetaceans and raoellid artiodactyls [3]

Selon leur hôte préféré, leurs propriétés bactériologiques et leurs traits génétiques, trois groupes différents de *Brucella* marines ont été identifiés : type de dauphin *B. ceti,* type de marsouin *B. ceti* et type humain de *B. ceti.* Il semble que le type marsouin *B. ceti* soit plus étroitement lié aux isolats humains de *B. ceti* et au groupe de *B. pinnipedialis,* tandis que le type dauphin de *B. ceti* leur paraît ancestral. La phylogénie de *Brucella* ne semble pas refléter la phylogénie des hôtes préférés des espèces de ce genre bactérien [82]. Mais, sur la base d'une analyse phylogénétique comparative, qui indique que les pinnipèdes et les cétacés sont respectivement rattachés à l'ordre des carnivores et à la famille des Raoellidae, il est possible que l'ancêtre de *B. ceti* ait été hébergé par un hôte artiodactyle terrestre proche de cette famille il y a environ 58 millions d'années (Fig. 1) [3].

**Figure 2:**
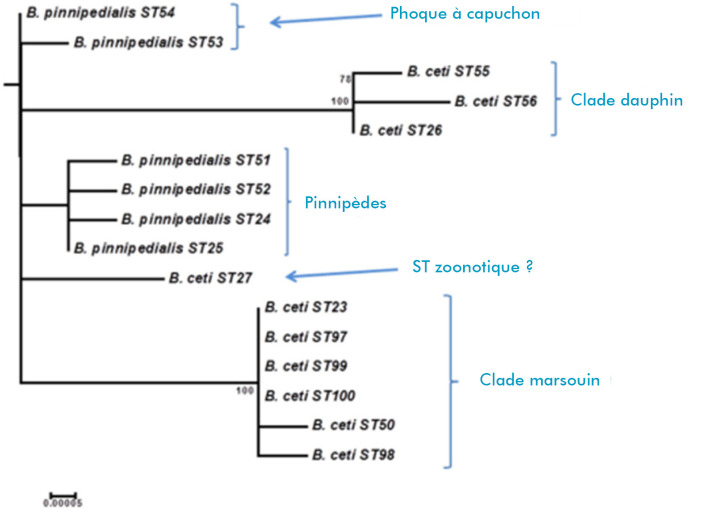
Relations phylogénétiques de *B. ceti* et *B. pinnipedialis* basées sur des données de séquences concaténées [85] *Phylogenetic relationships of* B. ceti *and* B. pinnipedialis *based on concatenated sequence data [85]*

Les types de séquence (ST) des 3 groupes de *Brucella* marine divergent en fonction de leur hôte d'origine. Ainsi, il existe trois grandes lignées de souches de mammifères marins de *Brucella* spp. : *Brucella ceti* ST23, que l'on trouve principalement chez les marsouins; *B. ceti* ST26 chez les delphinidés pélagiques et les ziphiidés; et *Brucella pinnipedialis* ST24/25 principalement chez les phoques. Les petits rorquals (*Balaenoptera acutorostrata*)*,* par contre, peuvent être infectés naturellement par des membres de toutes les principales lignées distinctes de *Brucella* associées aux mammifères marins [24]. Toutefois, la combinaison de techniques bactériologiques, moléculaires et de génotypage a permis d'isoler un autre génotype ST27 chez les humains, le grand dauphin (*Tursiops truncatus*) [13, 14], l'otarie de Californie (*Zalophus californianus*) [38, 84], le dauphin commun (*Delphinus delphis*) et le cachalot (*Physeter macrocephalus*) [13]. N’étant pas capnophile, le génotype ST27 responsable de quelques cas cliniques chez des personnes a été suggéré appartenir à *B. ceti,* mais d'autres caractéristiques indiquent que ce groupe pourrait être unique (Fig. 2) [85]. Des études ont indiqué que la pathogénicité ou le tropisme des organes varie selon le type de séquence (ST) de *B. ceti* chez les cétacés [13]. Dans les souches marines de *Brucella,* le man-BO-Ag porte un IS711, important pour la synthèse de pérosamine [86]. La séquence partielle du génome de *B. ceti* démontre la présence de gènes qui codent pour des facteurs virulents ou leur machinerie biosynthétique, comme la sécrétion de type IV VirB système, le système règlementaire BvrR/BvrS à deux composants, les β-glucanes, la protéine BacA, les composants de type flagelle, la phosphatidylcholine, ainsi que de nombreux autres gènes impliqués dans la survie intracellulaire de *Brucella abortus, B. melitensis* ou *B. suis* [72]. Les recherches en génétique sur la brucellose des cétacés peuvent aider à comprendre l’évolution et l'histoire naturelle de cette maladie et d'autres maladies infectieuses dans les populations migratrices marines de distribution mondiale n'ayant pas été vaccinées ni traitées avec des antibiotiques.

## Espèces réceptrices des souches de *Brucella* marines

La brucellose marine a été mise en évidence chez de nombreuses espèces de cétacés et de pinnipèdes [41], chez l'ours polaire (*Ursus maritimus*) [75] et les loutres d'Europe (*Lutra lutra*) [31] dont certaines populations vivent en mer le long des côtes, et ce via, soit l'isolement et la culture, soit la détection des anticorps. Toutefois, l'incidence réelle de la brucellose chez ces espèces reste difficile à établir étant donné le mode de vie de ces mammifères et leurs vastes répartitions.

Les espèces de cétacés chez lesquelles elle a été le plus souvent trouvée sont : le marsouin commun (*Phocoena phocoena*)

[26, 47, 69], le dauphin commun (*Delphinus delphis*) [19, 47, 73], le grand dauphin (*Tursiops truncatus*) [29, 47], le dauphin obscur (*Lagenorhynchus obscurus*) [79], le lagénorhynque à flanc blanc (*L. acutus*) [16], le dauphin bleu et blanc (*Stenella coeruleoalba*) [10.47], le marsouin de Burmeister (*Phocoena spinipinnis*) [79], le rorqual commun (*Balaenoptera physalus*) [78], le rorqual boréal (*Balaenoptera borealis*) [78], le petit rorqual (*Balaenoptera acutorostrata*) [10], la baleine à bec de Sowerby (*Mesoplodon bidens*) [34], le globicéphale noir (*Globicephala melas*) [21.34.47] et l'orque (*Orcinus orca*) [47].

Parmi les espèces de pinnipèdes réceptrices on peut citer : le phoque annelé (*Phoca hispida*) [30, 32, 43], le phoque à capuchon (*Cystophora cristata*) [62], le phoque gris (*Halichoerus grypus*) [31, 47, 69], le phoque du Groenland (*Pagophilus groenlandicus*) [30], le phoque commun du Pacifique (*Phoca vitulina richardii*) [35], le phoque commun (*Phoca vitulina*) [7, 48, 73], le phoque de Weddell (*Leptonychotes weddellii*) [70], l'otarie de Californie (*Zalophus californianus*) [40] et le morse de l'Atlantique (*Odobenus rosmarus rosmarus*) [58].

Par ailleurs, les helminthes [26, 68] ainsi que leurs réservoirs, les poissons [43] et les humains [55, 83] peuvent aussi héberger des souches de *Brucella* marines.

## Sensibilité des espèces aux souches de *Brucella* marines

Il est difficile de déterminer les signes cliniques et pathologiques directement liés aux infections des mammifères marins à *Brucella.* En effet, dans la plupart des cas, les souches ont été isolées à partir de cétacés échoués dans des conditions sanitaires précaires ou déjà morts et les signes cliniques, s'ils étaient présents, n'ont pas pu être observés. D'un autre côté, il n'est pas rare de trouver d'autres espèces bactériennes, fongiques ou parasitaires dans les mêmes organes et tissus à partir desquels *Brucella* a été isolée et de ce fait, le diagnostic clinique et pathologique différentiel de la brucellose chez les cétacés reste délicat [22, 25, 36].

### Cétacés odontocètes

Chez les cétacés odontocètes (à dents), plusieurs cas d'avortement ont été signalés chez des femelles infectées par *B. ceti* [29, 46, 57]. Aussi, quelques symptômes ont été décrits chez des dauphins associés à l'infection par *B. ceti* comme des difficultés à la nage et des problèmes d'orientation chez des dauphins présentant une neurobrucellose [22, 39, 41]. Les espèces les plus sensibles chez les cétacés odontocètes semblent être le marsouin commun, le dauphin bleu et blanc avec des signes cliniques graves associés à de sévères lésions de type méningo-encéphalite [43, 46], le grand dauphin *T. truncatus* et le dauphin commun *D. delphis.* Des lésions ont également été rapportées chez d'autres espèces de cétacés, telles que des lésions typiques de méningo-encéphalite lymphocytaire associées à *B. ceti* ST26 chez des baleines à bec de Sowerby mâles juvéniles *M. bidens* [20] et une méningo-encéphalite associée à *B. ceti* chez un globicéphale noir (*G. melas*) [21].

### Marsouin commun (*Phocoena phocoena*)

De graves lésions associées à *Brucella* au niveau de plusieurs organes tels que les poumons, le cœur, les articulations, la peau, le cerveau et les os ont été observées chez les marsouins communs. En effet, *B. ceti* a été isolée d'une discospondylite spinale non clinique [32] ainsi que de la colonne vertébrale et des vertèbres [53]. On l'a retrouvée dans les poumons d'un marsouin échoué présentant une infestation du ventricule droit du cœur et des vaisseaux sanguins pulmonaires par des nématodes associée à des thrombi pulmonaires et à une pneumonie nécrosante aiguë et à une pneumonie interstitielle subaiguë avec artérite [46]. Chez les marsouins communs, d'autres lésions à partir desquelles *Brucella* a été isolée ont été décrites telles qu'un abcès hépatique, une péritonite, une épididymite, une orchite [32] et des abcès multiloculaires au niveau des testicules [15]. Un avortement chez une femelle échouée sur la côte belge dû à une infection à *B. ceti* a été suspecté [46].

### Dauphin bleu et blanc (*Stenella coeruleoalba*)

Cette espèce est connue pour présenter le plus fréquemment des signes de neurobrucellose [22, 36, 41]. Les méninges apparaissent hyperhémiques et le volume du liquide céphalo-rachidien est augmenté [42]. Un dauphin bleu et blanc femelle a développé une endocardite sévère, très ressemblante aux lésions cardiaques que l'on peut observer chez des humains infectés par *Brucella* [39]. Cette endocardite est caractérisée par un nodule et de la fibrine sur la valvule mitrale. *B. ceti* a été aussi isolée de diverses lésions, chez des individus échoués à l’état vivant, en particulier du système nerveux central associée à une pathologie neurologique ainsi que de l'utérus, des testicules, des fœtus avortés, des sécrétions vaginales, du lait et des glandes mammaires [43]. Un mâle dauphin bleu et blanc a présenté une arthrite et une ostéite fibrino-purulente de l'articulation scapulo-humérale droite avec infiltration de la cavité synoviale par des macrophages et des polynucléaires neutrophiles [16]. Cet animal présentait aussi une neurobrucellose, microscopiquement méningo-encéphalomyélite. Des lésions pulmonaires ressemblant à celles observées chez les humains atteints de brucellose ont également été enregistrées chez cette espèce de dauphin. Ces lésions correspondent à une pneumonie interstitielle et à une bronchopneumonie, avec des microcalcifications bronchiolaires, une hyperémie et de petits agrégats de leucocytes dans le tissu conjonctif péribronchiolaire [39, 40].

### Grand dauphin (*Tursiops truncatus*)

Des cas de placentites et d'avortements dus à une infection à *Brucella* ont été documentés et la bactérie a été isolée à partir de plusieurs tissus de fœtus avortés chez des grands dauphins gardés dans des aquariums en Californie [29, 57]. Une dyspnée a été notée chez un grand dauphin présentant un abcès pulmonaire [9]. D'autres lésions telles que l’épaississement et la congestion des leptoméninges avec arthrite (séro) fibrino-suppurative et proliférative de l'articulation de l’épaule, associées à la détection de *Brucella* dans le tissu cérébral et dans les lésions articulaires du cerveau et de l’épaule, ont été notées chez cette espèce [74].

### Dauphin commun (*Delphinus delphis*)

Une méningite associée à une arthrite à *B. ceti* a été signalée chez le dauphin commun [19]. Un abcès sous-cutané à partir duquel *Brucella* a été isolée a été observé chez un individu de cette espèce [32]. Des lésions histologiques évoquant une neurobrucellose ont aussi été notées chez trois individus de cette espèce, associées à une détection de *Brucella ceti* dans leurs rates et dans le cerveau d'un individu [13].

### Globicéphale noir (*Globicephala melas*)

Une méningo-encéphalite associée à *B. ceti* chez un globicéphale noir a été enregistrée et une *Brucella* a été isolée, en plus grand nombre dans le liquide céphalo-rachidien que dans le tissu cérébral [21]. Chez cette même espèce, *B. ceti* a été isolée du testicule ce qui fournit encore une preuve supplémentaire de l'impact de cet agent pathogène sur le système reproducteur des cétacés [34].

### Cétacés mysticètes (à fanons)

Le petit rorqual semble être l'espèce de cétacé mysticète la plus sensible. Un petit rorqual commun (*B. acutorostrata*) de l'ouest du Pacifique Nord avec une sérologie positive a présenté plusieurs granulomes nodulaires dans l'endomètre utérin [64]. Ces lésions montraient une importante infiltration mononucléaire et des cellules épithélioïdes et géantes, suggérant une pathologie induite par une invasion brucellique. Un autre petit rorqual commun a présenté un grand abcès similaire aux lésions observées chez les odontocètes, en particulier chez des delphinidés pélagiques infectés par *B. ceti* (génotype ST26) [24]. Des orchites granulomateuses et parfois des abcès nécrotiques ont été trouvés chez des petits rorquals et des baleines de Bryde (*B. edeni*) mâles [63]. Une neurobrucellose a été enregistrée chez un petit rorqual commun, associée à l'isolement de *B. pinnipedialis* ST24 et de gamma-herpesvirus 2. Il s'agit du premier signalement de *B. pinnipedialis* qui pourrait être due à une co-infection avec un herpès, virus connu pour être en lien avec une immunosuppression [23].

### Pinnipèdes

Les pinnipèdes semblent peu sensibles à la brucellose car peu de signes cliniques sont décrits et les données sur les découvertes pathologiques en association avec *B. pinnipedialis* chez les phoques (*Phocidae*) sont très rares [41]. La plupart des isolements de *Brucella* à partir de tissus infectés proviennent d'animaux apparemment sains sans manifestations cliniques. Si une lésion est associée à l'isolement ou à l'identification de la bactérie, il s'agit principalement d'une bronchopneumonie d'origine vermineuse [43, 49]. Une *Brucella* spp. issue d'animaux de ferme a été détectée par PCR chez une otarie de Californie (*Z. californianus*)*,* cependant son espèce n'a pas pu être définitivement identifiée [2].

### Autres mammifères marins (ours polaires, loutres)

Des anticorps ont été trouvés dans des échantillons de plasma de 297 ours polaires (*U. maritimus*) du Svalbard et de la mer de Barents [77]. Une loutre de mer sauvage (*Enhydra lutris nereis*) du sud des côtes californiennes découverte avec ostéomyélite, arthrite, abcès sous-cutanés et signes neurologiques était vraisemblablement infectée par une *Brucella* de l'espèce *pinnipedialis.* Bien que le séquençage du gène *omp2a* ait révélé une homologie de 100 % avec les *Brucella* spp. infectant les pinnipèdes, les baleines et les humains, les séquences du gène *omp2b* n’étaient identiques qu'aux isolats d'origine pinnipède. Toutefois, le typage de séquence multilocus a classé l'isolat de loutre de mer comme ST26. Cet animal ayant été co-infecté par *Toxoplasma gondii*, les signes neurologiques observés seraient éventuellement causés par ce parasite [56].

### Les modèles animaux

Des infections expérimentales *in vivo* avec des *Brucella* de mammifères marins ont été réalisées avec diverses souches chez différentes espèces animales terrestres (souris BALB/c, cobayes, porcelets…) mais les résultats n'ont pas toujours été concluants. Les *B. ceti* de types dauphins et marsouins semblent afficher une faible infectiosité pour les humains et une virulence variable dans les modèles d'animaux terrestres [59, 60]. De plus, il a été démontré que des souches de *Brucella* de phoque étaient capables d'induire expérimentalement une séroconversion et des avortements chez le bétail, de façon moins sévère qu'avec *B. abortus* [71]. De jeunes truies non gravides ont été infectées momentanément par un isolat ST27 provenant d'un patient humain et des cobayes infectés par voie intramusculaire avec la *Brucella* d'un mammifère marin. Ces souches étaient moins virulentes que celles provenant d'animaux terrestres [59].

### Humains

Le premier cas de transmission zoonotique fut rapporté par Brew *et al.* (1999). Il concerne un chercheur qui manipulait des souches de *Brucella* isolées de mammifères marins [8]. Les symptômes qu'il présenta furent des céphalées, de la fatigue ainsi qu'une sinusite sévère. Le patient était devenu séropositif pour *Brucella* et la souche isolée était semblable à celle des mammifères marins. Whatmore *et al.* (2008) décrivent que cette infection en laboratoire a été provoquée par une séquence type ST23, un génotype la plupart du temps associé aux marsouins communs [83].

Trois cas d'infections naturelles ont également été rapportés. Deux de ces cas de brucellose causés par des souches de mammifères marins se sont présentés au Pérou. Ces individus consommaient des coquillages et crustacés crus mais n'avaient pas eu de contact avec des mammifères marins [74, 83]. Bien que ces 2 cas soient apparus à 15 ans d'intervalle, ils ont de nombreuses similitudes en ce qui concerne l’épidémiologie, les manifestations cliniques et l'histopathologie [75]. Ces deux patients présentaient une symptomatologie nerveuse. Le premier avait des douleurs périorbitaires, des céphalées ainsi que des crises tonico-cloniques régulières. Le second avait également des céphalées, ainsi que des nausées, des vomissements et des détériorations progressives de la vue [75]. Le troisième cas est survenu en Nouvelle-Zélande. Il n'avait pas eu de contact avec des mammifères marins non plus mais avait consommé du poisson cru [55]. L'examen par résonance magnétique de ce dernier patient a mis en évidence des inflammations multifocales entreprenant les vertèbres lombaires 1 et 4 ainsi que l'ilium droit. Ces trois cas cliniques semblent être liés à l'espèce *B. ceti* et au génotype ST27 [41].

## Sources de contamination

Certains auteurs suggèrent que l'océan ne serait pas une source possible de *Brucella* et ce en raison de l'effet de dilution qui compromet fortement la rencontre entre les *Brucella* non mobiles et leurs hôtes et aussi des conditions environnementales qui sont souvent extrêmes notamment en termes de pH, de température et d'osmolarité [41, 43, 62]. Le réservoir des *Brucella* marines serait représenté par toutes les espèces animales infectées qui peuvent excréter la bactérie [19]. Les carcasses des mammifères marins échoués, restant parfois sur place pendant de longues périodes, pourraient être une source de transmission aux animaux marins sauvages comme d’élevage, en particulier pour ceux qui sont proches des bandes côtières. Les poissons seraient aussi porteurs de *Brucella* et pourraient la transmettre aux mammifères marins.

Les organes qui véhiculent *Brucella* sont essentiellement les poumons, les organes reproducteurs, les glandes mammaires, ainsi que les vers parasites. En effet, des placentites et des avortements dus à une infection à *Brucella* ont été documentés et la bactérie a été isolée à partir de plusieurs tissus d'avortons [29, 57]. *Brucella* a également été isolée des glandes mammaires des cachalots et des dauphins, suggérant une invasion de macrophages résidents dans ces organes [32, 39], si bien que le lait de dauphin (*S. coeruleoalba*) pourrait être une source d'infection pour le nouveau-né ou les humains en contact étroit [42]. En ce qui concerne les vers parasites, dès 1997, Garner et ses collaborateurs ont pu identifier la présence de *Brucella* spp. dans l'utérus et l'appareil digestif d'un nématode pulmonaire femelle, *Parafilaroides,* isolé chez un phoque commun (*P. vitulina*) [35]. La présence de bactéries immuno-marquées dans des granulomes inflammatoires pulmonaires contenant des larves de nématode et la présence d'antigènes de *Brucella* dans les macrophages du parenchyme pulmonaire à proximité des parasites infectés suggèrent que les vers pulmonaires pourraient constituer une source de transmission de *B. pinnipedialis* au phoque commun [66]. Cette hypothèse a été appuyée par d'autres études plus récentes chez les cétacés qui ont signalé que des nématodes pulmonaires des genres *Parafilaroides, Halocercus* et *Pseudalius* parasitant des dauphins et des marsouins infectés peuvent contenir des quantités relativement importantes de *Brucella* [25, 46, 69]. Certaines études suggèrent même que dans 50 % des cas, il y a association de *Brucella* et d'une infestation parasitaire pulmonaire [49]. D'autres tissus desquels *Brucella* a été isolée peuvent aussi constituer une source d'infection. Ainsi, cette bactérie a été isolée d'ulcères cutanés et d'abcès sous-cutanés de certaines espèces de mammifères marins [41, 43, 46]. Par ailleurs, *B. pinnipedialis* a été mise en évidence dans la salive et l'urine de phoques communs [49].

## Modes de transmission

De manière générale, les voies de transmission de la brucellose sont encore moins comprises chez les pinnipèdes que chez les cétacés [43]. Chez ces derniers, on retrouve la plupart des modes de transmission connus pour les animaux terrestres. Ainsi, les rapports sexuels, l'alimentation maternelle, les fœtus avortés, les tissus placentaires, la transmission verticale de la mère au fœtus constituent les modes de transmission les plus probables de *B. ceti* [32, 39, 41]. De plus, certains vers parasites semblent être capables de traverser le placenta puis d’être transmis de la mère au fœtus [18]. Contrairement aux cétacés qui sont entièrement aquatiques, les pinnipèdes passent au moins leur période de reproduction à terre et en groupes formant des structures sociales proches, favorisant ainsi des modes de transmission directe et indirecte [49]. L'infestation par les parasites se ferait via l'ingestion des poissons parasités, et la séroprévalence des jeunes mammifères marins augmenterait brutalement juste après le sevrage impliquant une transmission possible par ces proies [49]. La réceptivité des poissons aux *Brucella* marines a été étudiée à l’échelle expérimentale. Il a été montré qu'une souche de *B. pinnipedialis,* isolée d'un phoque à capuchon, a survécu chez la morue de l'Atlantique (*Gadus morhua*)*,* ce qui indique que ce poisson pourrait jouer un rôle dans la transmission de cet agent pathogène aux phoques [61]. D'un autre côté, *Brucella melitensis* (biovar 3) a été isolée des organes internes et de prélèvements cutanés de poisson-chat du Nil (*Clarias gariepinus*) séropositif, naturellement infecté dans la région du delta du Nil en Égypte. Les déchets animaux étant généralement déposés dans les canaux d'eau de la région, le fleuve pourrait avoir été contaminé par des brucelles mais on ne sait pas quel rôle les poissons pourraient avoir dans la transmission en tant que réservoirs d'infection dans les milieux aquatiques [28]. Comme les cétacés, les pinnipèdes seraient infectés par les vers pulmonaires, à travers la consommation d'espèces de poissons hôtes intermédiaires dans ce cas. Ainsi, le parasite *Parafilaroides decorus* chez l'otarie de Californie utilise un poisson côtier présent dans la zone de balancement des marées, une girelle (*Girella nigricans*)*,* comme hôte vertébré intermédiaire [17] tandis que les larves des vers *Pseudalius gymnurus* et *Pseudalius inflexus* ont été isolées de la plie (*Platessa pleuronectes*) et de la limande (*Limanda limanda*)*.* Les mammifères marins infectés par ces parasites peuvent aussi servir de proie à différentes espèces prédatrices tant marines que terrestres, y compris les humains. La transmission par la consommation de proies est également étoffée par la séroprévalence non nulle des ours polaires et des orques, consommateurs de phoques potentiellement infectés [32, 49, 77].

Les *Brucella* pourraient être transmises des animaux marins aux humains par contact direct [8] avec des individus infectés, ou par ingestion de produits alimentaires infectés [55, 75, 83] et inhalation d'aérosols.

## Facteurs d'exposition

Dans le cas des animaux, des études portant sur un nombre limité d'individus ont prouvé que les xénobiotiques favorisent l'exposition des mammifères marins à la brucellose. Ainsi, Davidson et ses collaborateurs (2011) ont montré que l'infection par *Brucella ceti* est associée à la présence d'un haut taux de chlorobiphényl dans la graisse de dauphins [25]. Une étude sur l’épidémiologie de *B. pinnipedialis* chez les phoques communs (*P. vitulina*) vivants, capturés et échoués de 1994 à 2006 a montré d'une part l'absence d'une prédisposition sexuelle dans l'exposition ou l'infection par *B. pinnipedialis,* et d'autre part la présence d'une différence significative de prévalence entre les classes d’âge, les animaux sevrés, les jeunes d'un an et les subadultes étant les plus exposés et les plus infectés [49]. Comme déjà évoqué, les dauphins et les pinnipèdes présentant une pneumonie vermineuse semblent être plus prédisposés à l'infection par *Brucella* [25, 49, 66]. Toutefois, l'exposition des mammifères terrestres à des souches de *Brucella* via les parasites des poumons des cétacés paraît inconcevable, sachant en plus que ces bactéries n'ont aucun tropisme particulier pour les poumons de ces animaux terrestres [4].

Dans le cas des humains, l'augmentation des contacts avec les cétacés dans le monde [7, 42, 51] augmenterait le risque de transmission de brucelles pathogènes de ces mammifères marins aux humains et aux animaux domestiques. Les groupes à risque seraient « les personnes qui travaillent dans des centres de réhabilitation ou d'exposition de mammifères marins, ainsi que toute personne qui s'approche d'un animal échoué ou d'une carcasse » [54, 59]. Comme *B. pinnipedialis* a été cultivée ou détectée par PCR à partir de la glande salivaire, des poumons, de la vessie et des matières fécales des phoques, les professionnels de la faune travaillant avec des phoques vivants infectés pourraient être exposés à la bactérie via les sécrétions orales, l'urine ou les matières fécales [49].

Les risques de contamination de l'humain par *B. ceti* et *B. pinnipedialis* sont encore incertains. En effet, le nombre d’études publiées sur ce sujet est très limité et il n'y a pas eu d’étude sur la détection des maladies évocatrices de brucellose ou des anticorps dirigés contre les brucelles chez les personnes exposées professionnellement aux mammifères marins. Néanmoins, les données disponibles et résumées ici n’écartent pas le risque zoonotique.

## Conclusion

La brucellose chez les mammifères marins, découverte à la fin du xx^e^ siècle, a déjà fait l'objet d'un certain nombre d'observations et de publications dans la littérature scientifique. Malgré l'absence de l'estimation précise de la prévalence de cette maladie au sein des populations animales concernées, sa gravité semble avérée bien que le nombre de cas décrits, chez les mammifères marins (cétacés, pinnipèdes) comme chez les humains, reste modeste. *Brucella ceti* et *Brucella pinnipedialis* sont les deux espèces reconnues à l'heure actuelle, mais les données disponibles à leur sujet sont encore peu nombreuses. Les pinnipèdes semblent être des porteurs sains [50] mais sont aussi des porteurs chroniques de la bactérie (persistance dans des macrophages tissulaires) tandis que les cétacés peuvent développer diverses lésions de type méningite, orchite ou placentite avec avortements.

Les infections humaines dues à des *Brucella* spp. marines n'ont été suspectées que dans quelques cas. À l'heure actuelle, le véritable pouvoir pathogène des brucelles chez les mammifères marins et les humains n'est pas assez connu. Par analogie avec d'autres germes du genre *Brucella* responsables d'avortement chez les ruminants et de diverses pathologies dans l'espèce humaine, des mesures de prévention s'imposent.

Les voies de transmission de cette maladie n'ont pas encore été clairement établies mais il a été suggéré qu'outre la voie directe, des vers pulmonaires seraient impliqués dans la transmission et que des poissons puissent être des hôtes intermédiaires. Il en est de même quant à la contamination des humains, chez lesquels la voie d'infection n'est pas connue. Des travaux supplémentaires sont nécessaires pour identifier d’éventuels autres vertébrés et invertébrés qui pourraient être impliqués dans la transmission de ces bactéries.

Les risques zoonotiques de cette maladie, ainsi que l'augmentation significative du nombre d’échouages couplée à une haute séroprévalence chez certaines espèces de mammifères marins, doivent être pris en compte pour les personnes en contact direct ou indirect avec des mammifères marins. L’évaluation de ces risques nécessite une épidémio-surveillance continue associée à des examens post-mortem approfondis de toutes les espèces de mammifères marins échoués. Ces derniers sont considérés comme les sentinelles des environnements marins et côtiers car la grande mobilité des mammifères marins, que ce soit au large ou au niveau des côtes, les rend pertinents pour aborder de telles études. La recherche sur la brucellose des cétacés peut en outre aider à comprendre l’évolution et l'histoire naturelle de cette maladie et d'autres maladies infectieuses dans des populations mobiles et parfois migratrices, de distribution mondiale pour certaines, ainsi que la virulence et le développement de la pathogenèse dans une communauté animale qui n'a été ni vaccinée ni traitée avec des antibiotiques.

## Contribution des autrices

Les deux autrices ont contribué à l’élaboration du manuscrit

Hedia ATTIA EL HILI a assuré la soumission et le suivi des corrections

## Liens d'intérêts

Les autrices ne déclarent aucun conflit d'intérêts.
